# Impact of device programming on the success of the first anti-tachycardia pacing therapy: An anonymized large-scale study

**DOI:** 10.1371/journal.pone.0219533

**Published:** 2019-08-08

**Authors:** Saeed Shakibfar, Oswin Krause, Casper Lund-Andersen, Filip Strycko, Jonas Moll, Tariq Osman Andersen, Helen Høgh Petersen, Jesper Hastrup Svendsen, Christian Igel

**Affiliations:** 1 Department of Computer Science, University of Copenhagen, Copenhagen, Denmark; 2 Department of Cardiology, The Heart Centre, Rigshospitalet, University of Copenhagen, Copenhagen, Denmark; 3 Department of Clinical Medicine, Faculty of Health and Medical Sciences, University of Copenhagen, Copenhagen, Denmark; Universiteit Gent, BELGIUM

## Abstract

**Background:**

Antitachycardia pacing (ATP) is an effective treatment for ventricular tachycardia (VT). We evaluated the efficacy of different ATP programs based on a large remote monitoring data set from patients with implantable cardioverter-defibrillators (ICDs).

**Methods:**

A dataset from 18,679 ICD patients was used to evaluate the first delivered ATP treatment. We considered all device programs that were used for at least 50 patients, leaving us with 7 different programs and a total of 32,045 episodes. We used the two-proportions z-test (α = 0.01) to compare the probability of success and the probability for acceleration in each group with the corresponding values of the default setting.

**Results:**

Overall, the first ATP treatment terminated in 78.4%–97.5% of episodes with slow VT and 81.5%–91.1% of episodes with fast VT. The default setting of the ATP programs with the number of sequences S = 3 was applied to treat 30.1% of the slow and 36.6% of the fast episodes. Reducing the maximum number of sequences to S = 2 decreased the success rate for slow VT (*P* < 0.0001, *h* = 0.38), while the setting S = 4 resulted in the highest success rate of 97.5% (*P < 0*.*0001*, *h* = 0.27).

**Conclusion:**

While the default programs performed well, we found that increasing the number of sequences from 3 to 4 was a promising option to improve the overall ATP performance.

## Introduction

Implantable cardioverter-defibrillators (ICDs) are recommended in patients with various types of heart diseases in order to prevent sudden death and prolong life [1–3]. Antitachycardia pacing (ATP) automatically applied by implantable cardioverter-defibrillators (ICDs) effectively and painlessly terminates ventricular tachycardia (VT) with low risk of acceleration [4–6]. The type and parameters of the pacing are programmable; however, in practice, the default values are often used. The first treatment is often a burst. A burst consists of several sequences of pulse trains. In the default programming, three sequences are delivered, where each sequence consists of eight impulses with a constant coupling interval of 88% (i.e., the cycle length is 88% of the tachycardia cycle length) [4, 7].

The goal of this study was to evaluate the efficacy of different ATP programs and find possible options for more effective default programming of the devices. Since ATP is very effective, it is difficult to reliably measure differences between programs on small sample sizes. Furthermore, due to a large number of parameters important for device programming and the complex interaction between parameters, it is challenging to systematically assess the efficacy of different settings. Thus, instead of looking at a few patients and parameter variations in a clinical trial, we analyzed remote monitoring data from unselected patients. This allowed us to consider many patients and VT episodes and to focus on programming that is used in routine clinical practice.

The downside of our approach is that we had to base our study on anonymized data. Thus, we could not include possible confounding variables in our analysis, and therefore, the results should be validated independently in medical trials.

## Method

### Antitachycardia pacing

**Fig 1:**
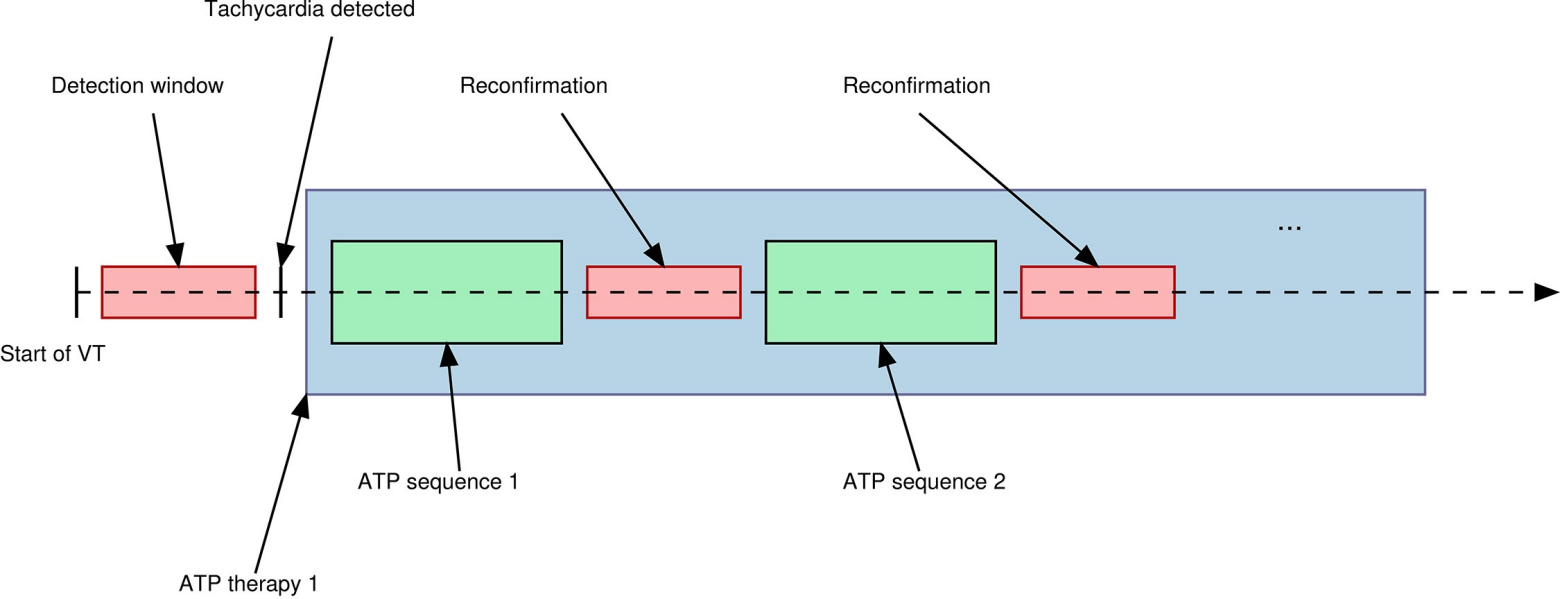
Schema of an ATP therapy.

In the following, we briefly describe the considered ATP therapy delivered by the ICD. The device uses electrical leads to monitor heart rhythm by observing features such as the cycle length, which is the time between two heartbeats, and the heart rate regularity. If a VT or ventricular fibrillation (VF) is detected, a series of ATP therapies are provided. Each ATP therapy consists of ATP sequences, as shown in Fig 1.

**Fig 2:**
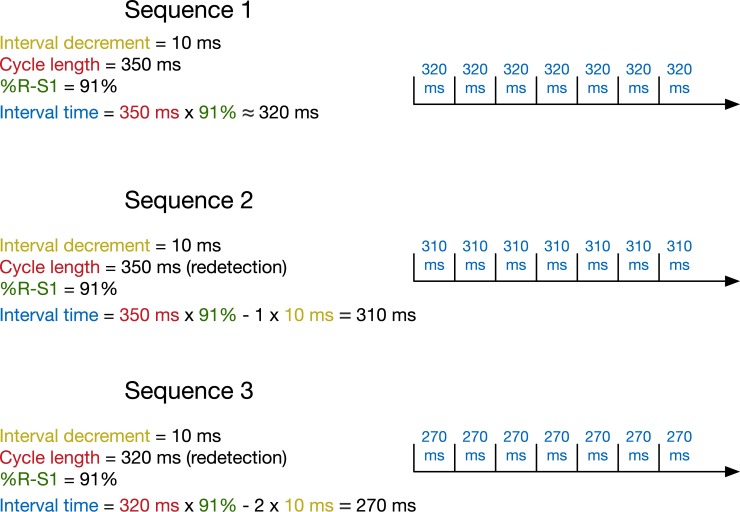
Schema of a burst ATP therapy. Shown are three sequences, with P = 7 pulses. The interval time between pulses is given by the detected cycle length times the coupling interval (%R-S1) minus an interval decrement for each repetition.

In this study, we focus on the ATP type burst, which is illustrated in Fig 2. Each ATP sequence consists of several (typically 6–10) stimuli (pulses). The time between the two pulses is called the interval time. The interval time is given by the detected cycle length multiplied by a factor called the coupling interval, which we refer to as %R-S1 in accordance with the documentation of the devices considered in this study. After each ATP sequence, it is checked if the VT/VF has been terminated. If not (i.e., in case of a re-detection), treatment continues based on the settings of the ICD, for example, by giving another ATP sequence.

In the burst setting, the newly computed interval time is decreased by a fixed Interval decrement with every sequence, see Fig 2 for an example.

### Study population

This study is based on data from 18,679 ICDs (Medtronic) implanted in the U.S.A. in the period ranging from 2005 to 2016 [8]. The device data were collected in the de-identified Medtronic DiscoveryLink database, and all patients consented to the use of their data for research purposes. Data from the same population were used for developing a machine learning algorithm for predicting electrical storm by Shakibfar et al. [8].

**Fig 3:**
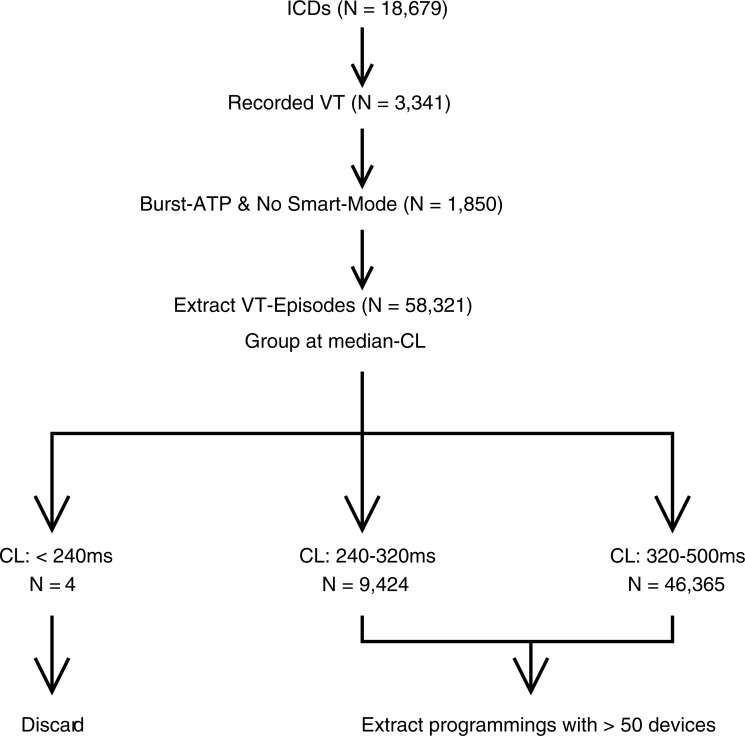
Chart of dataset generation.

Among all ICD patients, 3,341 ICDs recorded at least one VT. For each VT episode detected, we looked at the first delivered ATP treatment, which was in almost all cases of the type burst (> 95%), so other ATP types were discarded. Episodes, where the SmartMode option was activated, were also discarded because this setting changes the treatment based on previous success (causing a problem for proper statistical evaluation). The remaining 58,321 episodes from 1,850 devices were grouped by the median cycle length into slow VT (320–500ms, 46,365 episodes), fast VT (240–320ms, 9,424 episodes), and VF (< 240 ms, 4 episodes) [4, 6, 9]. The VF group was excluded from the analysis due to the small sample size. See Fig 3 for an illustration.

We considered all device programs that were used for at least 50 patients, leaving us with seven different programs and a total of 32,045 episodes.

The variable parameters were the coupling interval (%R-S1), the number of stimuli/pulses per burst (P), and the maximum number of sequences/bursts delivered to treat the VT (S). All programs used a cycle length interval decrement of 10 ms between sequences.

### Statistical evaluation

We counted the episodes where the first ATP treatment successfully terminated VT and those where the treatment accelerated the heart rate. Termination and acceleration were assessed by the device after remeasuring the median cycle length. We used the two-proportions z-test (α = 0.01)to compare the probability of success and the probability of acceleration in each group with the corresponding values of the default setting %R-S1 = 88, S = 3, and P = 8. Large sample sizes allow detecting very small differences between distributions with high probability. However, very small differences may not be important in practice although they are statistically significant. The relevance of the differences can be quantified by measuring the effect size, for example, using Cohen’s *h* [10]. To exclude irrelevant effects, we do not discuss significant differences with an effect size below *h* = 0.2 [10], which is the common threshold for small effect size.

## Results

**Fig 4:**
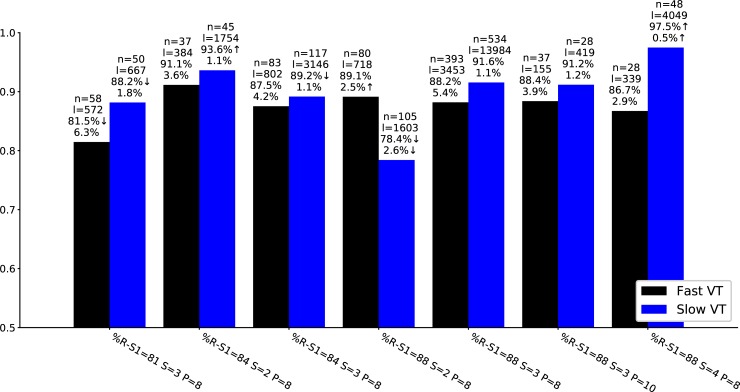
The fraction of terminated episodes for the different parameter settings for fast and slow VT. The settings differ in the coupling interval (%R-S1), a maximum number of sequences (S), and the number of pulses (P). The numbers above the bars are from top to bottom: number of patients (n), number of episodes (l), percentage of episodes where the first ATP treatment terminated VT, and the probability of acceleration. Results significantly better than %R-S1 = 88, S = 3, and P = 8 (*P*<0.01) are marked by ↑, significantly worse results are marked by ↓, and no arrow indicates non-significant differences compared to %R-S1 = 88, S = 3, and P = 8.

The seven types of programs considered and their success rates for treating slow and fast VTs for a given setting of the device parameters are given in Fig 4. Overall, the first ATP treatment terminated in 78.4%–97.5% of episodes with slow VT and in 81.5%–91.1% of episodes with fast VT.

The default setting %R-S1 = 88, S = 3, P = 8 was applied to treat 30.1% of the slow and 36.6% of the fast episodes. Using more pulses (P = 10) in this setting had no effect. However, reducing the maximum number of sequences to S = 2 decreased the success rate for slow VT (*P* < 0.0001, *h* = 0.38), while the setting S = 4 resulted in the highest success rate of 97.5% (*P* < 0.0001, h = 0.27). In contrast and somewhat surprisingly, for %R-S1 = 84, increasing the number of sequences from 2 to 3 did not lead to an improvement.

While %R-S1 = 81, S = 3, P = 8 was significantly worse in terminating VT in slow and fast episodes (*P* < 0.0001), the effect sizes when reducing %R-S1 were very small (*h* much smaller than 0.2).

## Discussion

The overall success rates of terminating VT with ATP were slightly higher than those reported in the literature [4, 6]. As pointed out by Wathen [6], one reason for a higher success rate is a larger S, which was, for example, set to S = 1 and S = 2 in the PainFREE Rx I and Rx II studies, respectively [4, 6]. The probability of acceleration ranged from 0.5% to 6.3% with fast VTs having higher acceleration risks.

There are limitations to this kind of large-scale studies. There is a lack of information on the clinical characteristics of the patients studied. Device-based interpretation of ATP success was used, no manual verification was performed. ICD therapy is initiated so quickly that it is impossible to separate an arrhythmia into a sustained (duration 30 seconds or more) vs. a non-sustained arrhythmia (duration less than 30 seconds). This precludes the consideration of the inappropriate ATP due to SVT or spontaneous termination of VT irrespective of applied ATP. Although one would assume that inappropriate ATP and spontaneous VT termination are equally distributed among the groups, they may play a role in smaller subgroups.

Since ICDs can deliver inappropriate therapy (often given to atrial fibrillation), assurance of correct arrhythmia diagnosis with the verification of ventricular arrhythmia is desirable. We must assume that a limited number of the episodes in this study were inappropriately diagnosed as VT/VF. All patients in the study were US patients, which may have influenced the strategy of device programming [11].

In conclusion, the default programming performed well. Increasing the number of sequences from 3 to 4 at %R-S1 = 88 and P = 8 increased the probability of terminating a slow VT episode without increasing the risk of acceleration or worsening the performance for fast episodes. Higher pacing frequencies (%R-S1 = 81) were not beneficial. These findings should be confirmed and further studied in clinical trials.

## References

[1] Ponikowski P, Voors AA, Anker SD, Bueno H, Cleland JG, Coats AJ, et al 2016 ESC Guidelines for the diagnosis and treatment of acute and chronic heart failure: The Task Force for the diagnosis and treatment of acute and chronic heart failure of the European Society of Cardiology (ESC)Developed with the special contribution of the Heart Failure Association (HFA) of the ESC. Eur Heart J. 2016;37(27):2129–200. 10.1093/eurheartj/ehw128 . 27206819

[2] Yancy CW, Jessup M, Bozkurt B, Butler J, Casey DE Jr., Drazner MH, et al 2013 ACCF/AHA guideline for the management of heart failure: a report of the American College of Cardiology Foundation/American Heart Association Task Force on practice guidelines. Circulation. 2013;128(16):e240–327. 10.1161/CIR.0b013e31829e8776 . 23741058

[3] Chen TH, Wo HT, Chang PC, Wang CC, Wen MS, Chou CC. A meta-analysis of mortality in end-stage renal disease patients receiving implantable cardioverter defibrillators (ICDs). PLoS One. 2014;9(7):e99418 Epub 2014/07/19. 10.1371/journal.pone.0099418 25036181 PMC4103758

[4] Wathen MS, DeGroot PJ, Sweeney MO, Stark AJ, Otterness MF, Adkisson WO, et al Prospective randomized multicenter trial of empirical antitachycardia pacing versus shocks for spontaneous rapid ventricular tachycardia in patients with implantable cardioverter-defibrillators: Pacing Fast Ventricular Tachycardia Reduces Shock Therapies (PainFREE Rx II) trial results. Circulation. 2004;110(17):2591–6. 10.1161/01.CIR.0000145610.64014.E4 . 15492306

[5] Grimm W, Plachta E, Maisch B. Antitachycardia pacing for spontaneous rapid ventricular tachycardia in patients with prophylactic cardioverter-defibrillator therapy. Pacing and Clinical Electrophysiology. 2006;29(7):759–64. Epub 2006/08/04. 10.1111/j.1540-8159.2006.00431.x . 16884513

[6] Wathen M. Implantable cardioverter defibrillator shock reduction using new antitachycardia pacing therapies. American Heart Journal. 2007;153(4 Suppl):44–52. Epub 2007/03/31. 10.1016/j.ahj.2007.01.020 . 17394902

[7] Wilkoff BL, Williamson BD, Stern RS, Moore SL, Lu F, Lee SW, et al Strategic programming of detection and therapy parameters in implantable cardioverter-defibrillators reduces shocks in primary prevention patients: results from the PREPARE (Primary Prevention Parameters Evaluation) study. Journal of the American College of Cardiology. 2008;52(7):541–50. Epub 2008/08/09. 10.1016/j.jacc.2008.05.011 . 18687248

[8] Shakibfar S, Krause O, Lund-Andersen C, Aranda A, Moll J, Andersen TO, et al Predicting electrical storms by remote monitoring of implantable cardioverter-defibrillator patients using machine learning. EP Europace. 2019;21(2):268–74. 10.1093/europace/euy257 30508072

[9] Nauffal V, Zhang Y, Tanawuttiwat T, Blasco-Colmenares E, Rickard J, Marine JE, et al Clinical decision tool for CRT-P vs. CRT-D implantation: Findings from PROSE-ICD. PLoS One. 2017;12(4):e0175205 Epub 2017/04/08. 10.1371/journal.pone.0175205 28388657 PMC5384669

[10] Cohen J. Statistical power analysis for the behavioral sciences. 2nd ed Hillsdale, N.J.: L. Erlbaum Associates; 1988 xxi, 567

[11] Mansour F, Khairy P. Programming ICDs in the Modern Era beyond Out-of-the Box Settings. Pacing Clin Electrophysiol. 2011;34(4):506–20. 10.1111/j.1540-8159.2011.03037.x . 21303392

